# Applications and clinical trial landscape using Toll-like receptor agonists to reduce the toll of cancer

**DOI:** 10.1038/s41698-023-00364-1

**Published:** 2023-03-08

**Authors:** Christian Rolfo, Elisa Giovannetti, Pablo Martinez, Shannon McCue, Aung Naing

**Affiliations:** 1 Center for Thoracic Oncology, The Tisch Cancer Institute, Icahn School of Medicine at Mount Sinai, Mount Sinai Health System, New York, NY USA; 2 Department of Medical Oncology, VU University Medical Center, Amsterdam, The Netherlands; 3 Cancer Pharmacology Lab, AIRC Start-Up unit, Fondazione Pisana per la Scienza, Pisa, Italy; 4 Sumitomo Pharma Oncology, Inc, Cambridge, MA USA; 5 Department of Investigational Cancer Therapeutics, University of Texas MD Anderson Cancer Center, Houston, TX USA

**Keywords:** Molecular medicine, Oncology

## Abstract

Toll-like receptors (TLRs), which serve as a bridge between innate and adaptive immunity, may be viable treatment targets. TLRs are the first line of defense against microbes and activate signaling cascades that induce immune and inflammatory responses. Patients with “hot” versus “cold” tumors may respond more favorably to immune checkpoint inhibition, and through their downstream effects, TLR agonists have the potential to convert “cold tumors” into “hot tumors” making TLRs in combination with immune checkpoint inhibitors, potential targets for cancer therapies. Imiquimod is a topical TLR7 agonist, approved by the FDA for antiviral and skin cancer treatments. Other TLR adjuvants are used in several vaccines including Nu Thrax, Heplisav, T-VEC, and Cervarix. Many TLR agonists are currently in development as both monotherapy and in combination with immune checkpoint inhibitors. In this review, we describe the TLR agonists that are being evaluated clinically as new therapies for solid tumors.

## Introduction

Tumors evade host immune surveillance through a variety of mechanisms, including selecting less immunogenic clones and exploiting immune checkpoints (e.g. cytotoxic T-lymphocyte-associated protein 4 [CTLA4], programmed cell death protein-1 [PD-1]) to promote immunologic tolerance^1,2^. Tumor cell engagement of either CTLA4 or PD-1 leads to the downregulation of effector T-cell responses by blocking T-cell receptor co-stimulation and driving T-cell anergy, respectively. Immunotherapy, a treatment approach that involves harnessing and augmenting the host immune system to respond to and eliminate malignant cells, seeks to circumvent these tumor defenses.

Immunotherapy has revolutionized the treatment of cancer and includes vaccines, monoclonal antibodies, adoptive cell therapies, and immune checkpoint inhibitors (ICIs). ICIs targeting CTLA4 (ipilimumab), PD-1 (nivolumab, pembrolizumab, cemiplimab), and the PD-1 ligand programmed death-ligand 1 (PD-L1; atezolizumab, avelumab, durvalumab) are approved for the treatment of various solid tumors, including melanoma, non-small-cell lung cancer, and urothelial carcinoma^3,4^. Although ICIs represent a significant advance in the treatment of cancer, not all patients respond to available agents, and there have been reports of serious immune-related adverse events (AEs) and delayed toxicity^3,5,6^. Consequently, efforts are underway to identify biomarkers of response/safety to currently available agents, to develop immunotherapies that are effective in a broader range of patients, and to evaluate the potential of combination regimens to enhance the antitumor activity of ICIs^3,7,8^. As part of these efforts, Toll-like receptors (TLRs), which serve as a bridge between innate and adaptive immune responses, have been proposed as viable treatment targets, both as single therapies and in combination with ICIs. TLR agonists induce cytokine secretion, leading to activation of cytotoxic T lymphocytes (CTLs), resulting in an immune response that mediates inflammation and can reduce tumor burden^9,10^. In a melanoma mouse model, tumor volume was reduced by resiquimod (TLR7 agonist) and enhanced when mice were treated with resiquimod in combination with a PD-L1 blocker^11^. Similarly, MBS8 (TLR7/8 agonist) demonstrated anti-cancer activity, leading to the elimination of tumors in syngeneic mouse models^12^. Guretolimod (DSP-0509), a TLR 7 agonist under evaluation in a clinical study (NCT03416335), showed significant tumor reduction in mice^13^. In these mouse models, the adaptive immune response was initiated as evidenced by the generation of tumor specific CD8 + T cells. There has been a progressive interest to explore the role of TLRs in the treatment of cancer. This review provides an overview of the trials and TLR compounds in development.

## TLRS: an overview

**Fig. 1:**
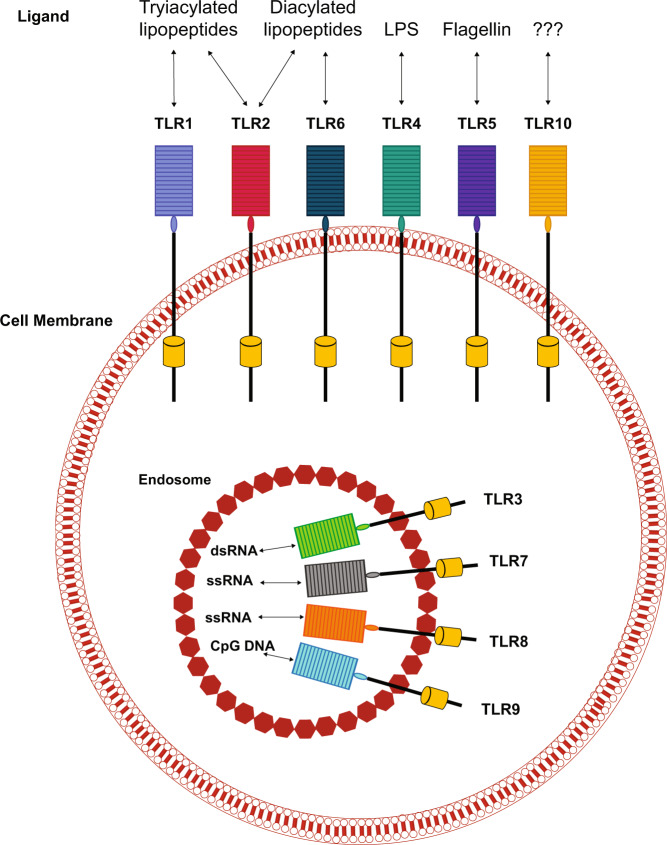
Cellular localization of the various TLRs and their prototypical ligands. The diagram illustrates the location of the TLRs. Dimerization of TLRs is required to activate downstream signaling but has not been shown.

TLRs are a family of transmembrane receptors expressed by various immune (e.g. macrophages, dendritic cells, lymphocytes) and non-immune (e.g. epithelial cells, fibroblasts) cells^14,15^. TLRs recognize conserved exogenous and endogenous danger signals known as pathogen-associated molecular patterns (PAMPs) and damage-associated molecular patterns (DAMPs), respectively^14,16^. PAMPs include the bacterial endotoxin lipopolysaccharide (LPS) and viral and bacterial nucleic acids^14,15^, while DAMPs are released by dead or dying host cells during programmed cell death processes^15,16^. Of the 10 TLRs expressed in humans, six are found on cell surfaces (TLR1, 2, 4, 5, 6, and 10), and four are localized to endosomes (TLR3, 7, 8, and 9; Fig. 1)^14,17^. The former recognizes proteins and lipids, whereas the latter engages nucleic acids^18^.

The binding of PAMPs or DAMPs to TLRs triggers the maturation and activation of antigen-presenting cells^14,19^. Once mature, macrophages and dendritic cells (1) secrete cytokines that stimulate pro-inflammatory responses and (2) present antigen to naive T lymphocytes, prompting their differentiation into effector T cells^17,20^. Through their critical roles in both innate and adaptive immunity, TLRs defend against invading pathogens and function in immune surveillance.

### Role of TLRs in cancer

TLRs have been shown to be overexpressed in different cancers, such as TLR7 and TLR8 in pancreatic cancer; TLR3, TLR4, TLR7, and TLR9 in esophageal cancer; TLR4, TLR5, and TLR7–9 in lung cancer; and TLR2–5 in ovarian cancer^18,21^. In a meta-analysis, overexpression of TLR1–5 or TLR9 was found to negatively correlate with clinical outcomes in patients with squamous cell carcinoma of the head and neck (SCCHN)^22^. In addition to overexpression, the localization of individual TLRs can become perturbed in malignant cells^23^. As mentioned, TLR2, TLR4, and TLR5 are normally localized to the plasma membrane, but can be found in the cytoplasm of colorectal cancer (CRC) cells. Similarly, TLR5 exhibits diffuse intracellular expression in esophageal carcinoma.

As a family, TLRs have been implicated in both cancer progression and suppression, with the effects of individual receptors varying by tumor histology^18,21,24–26^. For example, TLR4, whose principal ligand is LPS, promotes antitumor responses in hepatoblastoma, but pro-tumor responses in hepatocellular carcinoma and cervical cancer^25^. The antitumor effects of TLRs are mediated by the secretion of pro-inflammatory cytokines and the induction of tumor cell death, whereas their pro-tumor effects include facilitating cancer cell proliferation, survival, and metastasis, as well as immunosuppression^18,24,26^. TLRs can also stimulate regulatory T cells, which further contribute to the creation of a tumor-permissive immune environment^21,26^. The antithetical effects of TLRs have been attributed to variations in the response and expression of individual receptors by tumor cells and cells in the tumor microenvironment^18,19^. As a consequence, it is not possible to regard all TLRs and tumor types as equal; rather, it is necessary to parse out the role of a particular TLR in a given treatment setting, as some patients may derive greater clinical benefit from a TLR antagonist and others from a TLR agonist.

### Rationale for targeting TLRs in cancer

Pre-clinical and early clinical studies in solid tumors using TLRs therapies in development have shown antitumor activity. In healthy human donor whole blood, the TLR7/8 agonist, MBS8 showed induction of the cytokine, IFN-inducible protein-10 (IP-10), with low levels of tumor necrosis factor [TNF]-alpha (TNF-α), and interferon [IFN]-gamma (INF-γ) and demonstrated antitumor activity as a monotherapy and rescued anti-PD-1 resistance in mouse models^12^. Similar anti-tumor activity and resistance rescue results were found with the TLR7 agonists, resiquimod and guretolimod (DSP-0509)^11,13^.

To date, no TLR antagonist has received regulatory approval for the treatment of cancer. Two vaccines with adjuvants that contain TLR agonist components have been approved by the United States Food and Drug Administration (FDA)^14,18^. One is monophosphoryl lipid A (MPLA), a TLR4 ligand processed from the LPS of *Salmonella minnesota*^14,18^. MPLA is employed as an adjuvant in a prophylactic vaccine against human papillomavirus types 16 and 18, common causes of cervical cancer. As an adjuvant, MPLA enhances the antigen-presenting capabilities of macrophages and B cells, primes naive T cells, induces the maturation of dendritic cells, and stimulates antibody production^27^. The second TLR agonist approved for human use is bacillus Calmette-Guérin (BCG), a TLR2/4 agonist derived from a live attenuated variant of *Mycobacterium bovis*^14^. BCG was originally designed for use as a tuberculosis vaccine^14^, but as immunotherapy, BCG is administered intravesically to patients with non-muscle-invasive bladder cancer^14,18^. A third TLR agonist, imiquimod, has also been approved by the FDA. Imiquimod is a nucleoside analog that is applied topically to the skin of patients with superficial basal cell carcinoma. Imiquimod acts as ligand for TLR7^14,18^ which, in contrast to TLR2 and TLR4, is located intracellularly^14,17^. Upon engaging their respective receptors, BCG and imiquimod are believed to stimulate antitumor responses by stabilizing the antigen-presenting machinery of macrophages and dendritic cells^28–31^. These actions, in turn, lead to the secretion of pro-inflammatory cytokines (e.g. interleukin [IL]-2, TNF-α, INF-γ), and the activation of effector T cells that subsequently infiltrate tumors.

Therefore, through their downstream effects, TLR agonists have the potential to convert “cold tumors” into “hot tumors” characterized by intense immunologic activity. It has been suggested that patients with “hot tumors” respond more favorably to immune checkpoint inhibition than those with “cold tumors”^32^.

Considering the clinical success of MPLA, BCG, and imiquimod and the potential to augment the clinical efficacy of existing immunotherapeutic agents, novel TLR agonists are being explored as monotherapy, as part of combination therapy, and as vaccine adjuvants in patients with a variety of solid tumors, as described in detail below.

## TLR agonists in clinical development

### agonists of cell surface-expressed TLRs

Most TLR agonists in clinical development for the treatment of solid tumors target intracellularly expressed receptors. However, synthetic ligands for cell surface-expressed TLR4 and TLR5 are being evaluated.

#### TLR4

A phase 1 study of the intravenously administered TLR4 agonist GSK1795091 in combination with other immunotherapies including pembrolizumab, in adults with advanced solid tumors, has been completed (NCT03447314), however due to changes in manufacturing during the study, differences in biological activity occurred^33^. In the study, GSK1795091 was used with a combination partner of immunotherapy; 54 patients were treated. Most patients (51/54 [94%]) experienced at least 1 Grade 1/2 TEAE. Events included chills 41% (*n* = 22), nausea 37% (*n* = 20), fatigue 35% (*n* = 19), anemia 26% (*n* = 14), vomiting 22% (*n* = 12), decreased appetite 20% (*n* = 11), pyrexia and headache, each 15% (*n* = 8), weight decreased, dizziness and hypertension each 13% (*n* = 7) and constipation, diarrhea, arthralgia, and back pain, each 11% (*n* = 6). Events ≥3 were experienced by 44% (*n* = 24) patients. These included: anemia 11% (*n* = 6) fatigue, back pain and hypertension, each 4% (*n* = 2) and constipation 2% (*n* = 1). Although the data are limited, 3 patients had a PR including 2 patients treated with GSK1795091 in combination with pembrolizumab, and 13 patients achieved stable disease (SD) including 1 patient treated with GSK1795091 in combination with pembrolizumab. A pharmacodynamic response was observed for IP-10, IL-10, IL-Ra, IL-6 and monocyte chemoattractant protein-1 (MCP-1) but was not observed for TNF-α^33^.

#### TLR5

Entolimod is a subcutaneously administered TLR5 agonist derived from *Salmonella* flagellin. In a phase 1 study (NCT01527136) of 26 patients with advanced malignancies who received entolimod daily for 2 weeks Grade 1/2 adverse events included: transient hypotension 62% (*n* = 16), hyperglycemia 54% (*n* = 14) and fever 50% (*n* = 13). There were 3 adverse events of grade ≥3: rigors and pyrexia 4% (*n* = 1), transaminitis 4% (*n* = 1), and hypotension 4% (*n* = 1)^34^. Entolimod induced the secretion of IL-6, IL-8, and IL-10 and decreased the numbers of immunosuppressive cells and cytokines. No tumor responses were observed. A randomized phase 2 study (NCT02715882) of entolimod in patients with colorectal cancer (CRC) was initiated in Russia in 2016^35^, but the status of this trial is unknown. Currently, there is no active clinical study of entolimod as an anticancer agent.

### Agonists of intracellular TLRs

Agents specific to TLR3 (polyinosinic-polycytidylic [poly-ICLC], rintatolimod, BO-112) and TLR8 (motolimod, SBT6050) are being actively investigated. In this article, our discussion focuses on novel agents targeting TLR7, TLR 7/8 and TLR9 (Table 1) as a comprehensive review regarding the studies for TLR 3 products has already been published^36^.

**Table 1:** Overview of TLR7 and TLR9 agonists in clinical development for the treatment of solid tumors.

Agent	Latest clinical study phase	Route of administration	Studied regimens	Tumor types under study	Study status	Identifier	Common toxicities
Investigational TLR7 agonists							
TQ-A3334	I/II	Oral	Monotherapy	NSCLC	Unknown	NCT04273815	Decreased lymphocyte, neutrophil, and white blood cell counts; headache^32^
			Combined with anti-VEGF				
SHR2150	I/II	Oral	Combined with chemotherapy and anti–PD-1 or anti-CD47	Solid tumors	Recruiting	NCT04588324	N/A
RO7119929	I	Oral	Monotherapy	HCC	Recruiting	NCT04338685	N/A
				Biliary tract cancer			
				Solid tumors with liver metastases			
DSP-0509	I/II	IV	Monotherapy	Solid tumors	Recruiting	NCT03416335	N/A
			Combined with pembrolizumab				
BNT411	I/IIa	IV	Monotherapy	Solid tumors	Recruiting	NCT04101357	N/A
			Combined with atezolizumab, carboplatin, and etoposide	ES-SCLC			
APR003	I	Oral	Monotherapy	CRC with liver metastases	Recruiting	NCT04645797	N/A
Investigational TLR7/8 agonists							
BDB001	I	IASC	Monotherapy	Solid tumors	Active, not recruiting	NCT03486301	Chills/rigor, fever, fatigue, nausea, pruritus^38^
			Combined with pembrolizumab				
	I		Combined with atezolizumab		Active, not recruiting	NCT04196530	
	II		Combined with atezolizumab and RT		Recruiting	NCT03915678	
	II		Monotherapy		Active, not recruiting	NCT04819373	
BDC-1001	I/II	IV	Monotherapy	HER2-positive solid tumors	Recruiting	NCT04278144	N/A
			Combined with nivolumab				
CV8102	I	IT	Monotherapy	Melanoma	Active, not recruiting	NCT03291002	Fatigue, fever, chills, headache^41^
			Combined with anti–PD-1	cSCC			
				SCCHN			
				ACC			
TransCon TLR7/8	I/II	IT	Monotherapy	Solid tumors	Recruiting	NCT04799054	ISRs^45^
			Combined with pembrolizumab				
MBS-8	I	IV	Monotherapy	Solid tumors	Recruiting	NCT04855435	N/A
BDB-018	I	IV	Monotherapy	Solid tumors	Recruiting	NCT04840394	N/A
Imiquimod (UGN-201)	I	Intravesical	Monotherapy	Urothelial cancer	Recruiting	NCT05055050	N/A
	I	Intravesical	Combined with UGN-301	Non-muscle Invasive Bladder Cancer	Recruiting	NCT05375903	
Investigational TLR9 agonists							
Lefitolimod (MGN1703)	I	SC	Combined with ipilimumab	Solid tumors	Active, not recruiting	NCT02668770	Flu-like symptoms, ISRs^50^
Tilsotolimod (IMO-2125)	Ib	IT	Combined with investigational ICIs ± nab-paclitaxel	SCCHN	Active, not recruiting	NCT04196283	Pyrexia, fatigue, chills, nausea, vomiting^52^
	II	IT	Combined with ipilimumab and nivolumab	Solid tumors	Active, not recruiting	NCT03865082	
	Ib	IT	Combined with ipilimumab and nivolumab	Solid tumors	Active, not recruiting	NCT04270864	
	II	ID	Monotherapy	Operable melanoma	Recruiting	NCT04126876	
CMP-001	Ib	IT	Monotherapy	Melanoma	Active, not recruiting	NCT02680184	Flu-like symptoms, ISRs^56^
			Combined with pembrolizumab	Melanoma			
	II	IT	Combined with cemiplimab	cSCC	Recruiting	NCT04916002	
				MCC			
				TNBC			
	II	IT	Combined with pembrolizumab	SCCHN	Active, not recruiting	NCT04633278	
	II	IT	Combined with nivolumab	Melanoma	Active, not recruiting	NCT04698187	
	II/III				Active, not recruiting	NCT04695977	
	I	IT	Combined with nivolumab, ipilimumab, and radiosurgery	CRC with liver metastases	Active, not recruiting	NCT03507699	
	II	IT/SC	Combined with pembrolizumab	Operable melanoma	Recruiting	NCT04708418	
	I/II	IT	Combined with an investigational ICI	Pancreatic cancer	Recruiting	NCT04387071	
				Non-melanoma solid tumors			
	II	IT	Combined with nivolumab	Melanoma	Active, not recruiting	NCT03618641	
	II	IT	Combined with nivolumab	Melanoma	Recruiting	NCT04401995	
	II	IT/SC	Combined with nivolumab	CRPC	Not yet recruiting	NCT05445609	
	II	IT/SC	Combined with RT	TNBC	Recruiting	NCT04807192	
SD-101	I	IT	Combined with nivolumab and RT	Pancreatic cancer	Active, not recruiting	NCT04050085	Flu-like symptoms, ISRs^62,64^
	II	IT	Combined with pembrolizumab	Breast cancer	Recruiting	NCT01042379	
	II	IT	Combined with pembrolizumab, iADT, and RT	Oligo-metastatic prostate cancer	Active, not recruiting	NCT03007732	
	I	IT	Combined with BMS986178	Solid tumors	Active, not recruiting	NCT03831295	
	I/Ib	hepatic artery infusion	Combined with nivolumab and ipilimumab	Uveal Melanoma	Recruiting	NCT04935229	
	I/II	Hepatic Artery Infusion	Combined with pembrolizumab or nivolumab and ipilimumab	Liver tumors	Recruiting	NCT05220722	
TAC-001	I/II	IV	Monotherapy	Solid tumors	Recruiting	NCT05399654	N/A

*ACC* adenoid cystic carcinoma, *CD47* cluster differentiation 47; *CRC* colorectal cancer, *CRPC* castrate-resistant prostate cancer, *cSCC* cutaneous squamous cell carcinoma; *ES-SCLC* extensive-stage small-cell lung cancer, *HCC* hepatocellular carcinoma, *HER2* human epidermal growth factor receptor 2, *iADT* intermittent androgen deprivation therapy, *IASC* immune-stimulating antibody conjugate, *ICI* immune checkpoint inhibitor, *ID* intradermal, *ISR* injection site reaction; *IT* intratumoral; *IV* intravenous, *MCC* Merkel cell carcinoma, *N/A* not available, *nab* nanoparticle albumin-bound, *NSCLC* non-small-cell lung cancer, *PD-1* programmed cell death protein-1, *RT,* radiotherapy, *SC* subcutaneous; S*CCHN,* squamous cell carcinoma of the head and neck, *TLR* Toll-like receptor; TNBC triple negative breast cancer; VEGF vascular endothelial growth factor.

#### TLR7

TLR7 primarily recognizes viral genetic material, specifically single-stranded RNA^17^. TQ-A3334 is an oral TLR7 agonist that has been shown to induce the production of pro-inflammatory cytokines, such as IFN-α and IP-10^37^. Although the status is unknown, there was a phase 1/2 study of TQ-A3334 being conducted (NCT04273815) for the treatment of non-small-cell lung cancer. In this study, TQ-A3334 tablets were being administered weekly either alone or in combination with the anti-vascular endothelial growth factor receptor inhibitor anlotinib. In a dose-ascending, phase 1a study of 42 healthy Chinese volunteers, a single dose of TQ-A3334 was tolerable, with no grade 4–5 AEs, serious AEs, or treatment-related discontinuations reported^37^. AEs occurred in 67% (28/42) of clinical trial participants administered TQ-A3334, with decreased lymphocyte 50% (*n* = 21), neutrophil 29% (*n* = 12), and white blood cell counts 26% (*n* = 11) and headache (6% (*n* = 11) being the most frequent. The rates of the most common AEs were dose-dependent and generally resolved without intervention within 72 h of dosing. Nine (21%) grade 3 AEs were reported (decreased neutrophil count, 5% (*n* = 2); decreased lymphocyte count, 14% (*n* = 6); hypertriglyceridemia, 2% (*n* = 1)). Treatment-induced changes in cytokine expression also returned to baseline within 72 hours.

Intratumoral LHC165 (NCT03301896), was being explored as a single agent (*n* = 20) and in combination with the investigational PD-1 inhibitor spartalizumab (PDR001) (*n* = 19) in patients with advanced solid tumors. Treatment-emergent AEs were reported in 56% (22/39) of patients (all grades) and 5% of patients (*n* = 2) experienced ≥ grade 3 event^38^. The most frequent events in both the monotherapy and combination arms were pyrexia 10% (*n* = 2), and 26% (*n* = 5), injection site reaction 15% (*n* = 3) and 10.5% (*n* = 2), chills 5% (*n* = 1) and 10.5% (*n* = 2) and decreased appetite 10% (*n* = 2) and 5% (*n* = 1), respectively. In the combination arm 16% of patients (*n* = 3) reported pruritus and 10.5% (*n* = 2 each) reported asthenia, malaise, and vitiligo. One patient in the monotherapy arm reported 2 grade 3 events: neutropenia and lymphopenia and another patient in the combination reported a grade 3 event of pancreatitis. The best overall response for LHC165 showed that in the monotherapy arm 10% (*n* = 2) of patients had a partial response (PR) and in the LHC165 with spartalizumab arm, 5% (*n* = 1) had a PR and 21% (*n* = 4) had stable disease. Recently (Aug. 2022), the Sponsor terminated the study.

Intravenous BNT411 (NCT04101357) is being evaluated as monotherapy in patients with solid tumors and in combination with atezolizumab, carboplatin, and etoposide in those with chemotherapy-naïve, extensive-stage small-cell lung cancer. In 11 patients treated with monotherapy, drug-related Grade 1/2 AEs included: pyrexia 18% (*n* = 1), and anemia 18% (*n* = 2), and one patient reported a Grade 3 event of pyrexia. No dose limiting toxicities, serious AEs, or drug related grades 4 and 5 AEs reported. At the highest dose level tested, cytokine movement was observed with increases in IP10. Preliminary efficacy data included 1 patient who had a best response of SD for 5 months^39^.

Intravenous NJH395 is an immune-stimulating antibody conjugate (ISAC) that combines an unspecified TLR7 agonist with a monoclonal antibody against human epidermal growth factor receptor-2 (HER2)^40^. Despite its systemic distribution, the HER2 component directs the TLR7 agonist specifically to HER2-expressing cells, enabling it to act locally and theoretically limit its off-target effects. A phase 1 dose escalation study of NJH395 has been completed (NCT03696771) in individuals with non-breast, HER2-positive advanced malignancies. In an interim analysis performed on 18 patients who received a single infusion of NJH395, cytokine release syndrome 56% (*n* = 10), pyrexia 44% (*n* = 8), and nausea 44% (*n* = 8) were the most common AEs^40^. Grade ≥3 AEs included decreased lymphocyte counts 28% (*n* = 5) and increased aspartate aminotransferase 11% (*n* = 2). The authors described these toxicities as “significant, but manageable”. NJH395 was also shown to trigger an increase in the number of tumor-infiltrating cytotoxic T cells, and stable disease (SD) was the best observed tumor response in 50% (*n* = 9) patients at 3 weeks post-dose.

A phase 1 study (NCT04645797) of an orally administered TLR7 agonist, APR003, administered to patients with advanced CRC and metastatic liver lesions is recruiting. APR003 was designed to concentrate in the gastrointestinal tract and liver; this targeted distribution is anticipated to lead to a more favorable safety and tolerability profile relative to other agonists with a more systemic distribution^41^.

Several other first-in-human studies of TLR7 agonists are currently underway. These include oral SHR2150 (NCT04588324), which is being tested in combination with chemotherapy plus anti–PD-1 or anti-CD47 in patients with advanced solid tumors; oral RO7119929 (NCT04338685), which is being evaluated as a single agent in patients with advanced hepatocellular carcinoma, biliary tract cancer, or solid tumors with liver metastases. DSP-0509 (NCT03416335) administered intravenously, is being studied alone and in combination with pembrolizumab in adults with advanced solid tumors; this study is ongoing. Although clinical data is not yet available for these studies several other TLR7 agonists under study have reported some of the clinical data.

#### TLR7/8

Because of their shared phylogeny, it is not uncommon for agents that bind to TLR7 to also engage TLR8^42^. NKTR-262 is a TLR7/8 agonist in clinical development for the treatment of relapsed/refractory advanced solid tumors^43^. In patients with melanoma, intratumoral injection of NKTR-262 stimulated the up-regulation of IFN-inducible genes and IP-10 in a dose-dependent manner and increased the density of CD11c-positive cells; CD11c is expressed primarily on the surfaces of dendritic cells. In preclinical models, the antitumor activity of NKTR-262, which targets the innate immune system, was found to synergize with the investigational IL-2 pathway agonist bempegaldesleukin, which promotes the expansion of tumor-infiltrating cytotoxic T cells^44^. In the terminated phase 1/2 REVEAL study (NCT03435640), NKTR-262 was evaluated in combination with bempegaldesleukin ± nivolumab. In an interim analysis of phase 1, 97% (35/36) of patients receiving NKTR-262 plus bempegaldesleukin experienced ≥1 treatment-related AE, most commonly flu-like symptoms 78% (*n* = 8), fatigue 44% (*n* = 16), nausea 42% (*n* = 15), and pruritus 42% (*n* = )^43^. In 31% (*n* = 11) patients, Grade ≥3 AEs were reported in 6% (*n* = 2) patients each: elevated ALT, hypotension, leukocytosis, rash and syncope. A disease control rate of 41% (7/17 patients) including 2 with a partial response was also reported however, the Sponsor has terminated the study due to the overall Phase 1 results: but not due to safety reasons.

The intravenously administered TLR7/8 agonist BDB001 is being investigated in two ongoing phase 1 studies of patients with advanced solid tumors. In both, BDB001 will be administered as monotherapy and in combination with an immune checkpoint inhibitor, one with pembrolizumab (NCT03486301) and the other with atezolizumab (NCT04196530). Based on preliminary data from 36 patients participating in NCT03486301, the most common AEs following weekly administration of single-agent BDB001 were chills/rigor 19% (*n* = 7), fever 19% (*n* = 7), fatigue 11% (*n* = 4), nausea 11% (*n* =*n* = 4), and pruritus 11% (*n* = 4)^45^. Treatment-related AEs occurred in 70% (*n* = 25) patients. Two of the three patients with grade 3 AEs experienced cytokine release syndrome; no grade 4–5 AEs. BDB001 stimulated IFN-inducible genes, IFN-gamma, and IP-10 and the maturation of antigen-presenting cells. Efficacy data were available for 32 clinical trial participants, of whom 6% (*n* = 2) had a partial response (PR) and 56% (*n* = 20) had SD. A total of 23 patients with advanced solid tumors received BDB001 plus pembrolizumab in NCT03486301^46^. The safety profile of BDB001 when combined with pembrolizumab was largely similar to that observed with single-agent BDB001, with the most common treatment-related AEs being fever 39% (*n* = 9), fatigue 39% (*n* = 9)), chills/rigor 35% (*n* = 8), pruritus/rash 22% (*n* = 5), and nausea 13% (*n* = 3). Three treatment-related AEs were grade 3 (fatigue, rash, stomatitis, and alkaline phosphatase elevation); no grade 4–5 treatment-related AEs were reported. Among the 14 patients evaluated for efficacy, the disease control rate was 57% (*n* = 8). Two phase 2 studies of BDB001 are underway in combination with atezolizumab plus immunogenic radiotherapy in patients with solid tumors (NCT03915678; AGADIR) and as monotherapy in patients with solid tumors.

Like the TLR7 agonist NJH395, BDC-1001 is an ISAC. Although BDC-1001 also bears a HER2 monoclonal antibody, its TLR-binding component engages both TLR7 and TLR8^47^. In an ongoing, first-in-human, phase 1/2 study (NCT04278144), BDC-1001 is being studied as monotherapy and in combination with pembrolizumab in patients with HER2-positive advanced solid tumors. Interim data from the monotherapy cohort in this ongoing study demonstrated a favorable safety profile. In 57 patients the most frequent reported treatment-emergent AEs included: Grade 1 and 2 infusion-related reactions 19% (*n* = 11), pyrexia and diarrhea 8.8% (*n* = 5), fatigue 7% (*n* = 4), nausea and arthralgia 5.3% (*n* = 3), abdominal pain, anemia, and vomiting 1.8% (*n* = 1), and Grade ≥3 anemia 1.8% (*n* = 1)^47^.

CV8102 is a non-coding RNA sequence that activates innate immunity via TLR7/8 and the retinoic acid-inducible gene I pathway^48^. CV8102 is being explored as a single agent, administered via intratumoral injection, and in combination with a PD-1 antibody in a phase 1 study (NCT03291002) of patients with advanced melanoma, cutaneous squamous cell carcinoma (cSCC), SCCHN, or adenoid cystic carcinoma. In an interim analysis performed on 23 patients receiving CV8102 monotherapy and 13 patients receiving the doublet regimen, the most common AEs were fatigue, fever, chills, and headache. Grade 3 drug-related events (monotherapy cohort 17% (*n* = 4); and combination cohort 23% (*n* = 3)) occurred: increases in liver enzymes (*n* = 3), abscess at injection site (*n* = 1), hypotension (*n* = 1) and asymptomatic elevation of pancreatic enzymes (*n* = 2)^48^. In terms of antitumor efficacy, two objective responses, one complete and one partial, were observed in patients with melanoma receiving CV8102 monotherapy. Preliminary efficacy data included 3 patients treated with single agent CV8102 with SD for >6 months and regression of both injected and non-injected tumors, and 1 patient with a partial response (PR). In 2/25 patients treated in combination with an anti-PD-1 therapy 1 patient had a PR in the injected lesion but developed additional lesions and the other had a mixed response with regression in both injected and non-injected lesions but progression in other non-injected lesions^49^.

Intratumoral TransCon TLR7/8 Agonist is a prodrug of resiquimod^50,51^, a more potent derivative of the approved TLR7 agonist imiquimod^52^. To limit systemic effects, TransCon TLR7/8 Agonist was designed for intratumoral retention^50,51^. It is currently being evaluated as monotherapy and in combination with pembrolizumab in a first-in-human phase 1/2 study (NCT04799054) of patients with advanced solid tumors. Based on an interim analysis of eight clinical participants, TransCon TLR7/8 Agonist has yet to be associated with dose-limiting toxicities or treatment-related systemic AEs^53^. The only treatment-related AE reported to date has been transient, grade 1–2 injection site reactions.

#### TLR9

TLR9 recognizes unmethylated DNA sequences that are rich in phosphate-linked cytosine-guanine (CpG) dinucleotides^54^. Unmethylated CpG is common in bacterial and viral genomes, but rare in mammals, as their genomes are predominately methylated^54,55^. There are three classes of oligonucleotides^56^. Class A promotes the production and release of IFN-alpha by plasmacytoid dendritic cells and is a poor activator of B cells. The effects of Class B are converse to those of Class A, and Class C stimulates both plasmacytoid dendritic cells and B cells (ie, mix of Class A and Class B).

Lefitolimod (MGN1703), a double-stranded TLR9 agonist with a dumbbell-shaped structure^55^, is being evaluated in conjunction with ipilimumab in a dose-finding, phase 1 study (NCT02668770) of patients with advanced solid tumors. Per a company press release^57^, the decision to assess subcutaneous lefitolimod in combination with immunotherapy was made following disappointing topline results from the phase 3 IMPALA study (NCT02077868). In IMPALA, patients with metastatic CRC who responded to first-line chemotherapy were randomized to receive maintenance treatment of either twice-weekly lefitolimod or standard of care. In the primary analysis, median overall survival (OS) was 22.0 months among lefitolimod-treated patients versus 21.9 months among those receiving standard of care (hazard ratio [HR], 1.12; 95% confidence interval [CI], 0.91–1.38; *p* = 0.28), and median progression-free survival (PFS) was poorer for lefitolimod versus standard of care (data values not reported). Regarding safety, the press release only stated that “no new safety signals were detected”. In the phase 2 IMPACT study (NCT01208194), which compared maintenance lefitolimod (*n* = 43) with placebo (*n* = 13) in the same patient population, the most common treatment-related AEs were flu-like symptoms (lefitolimod 14% (*n* = 6); placebo 8% (*n* = 1)) and injection site reactions (lefitolimod 5% (*n* = 2); placebo 8% (*n* = 1)); the only grade 3–4 AE to occur in more than two patients was ileus 9% (*n* = 4), and no treatment-related serious AEs were reported^58^. Of note, in pre-planned biomarker analyses, higher (≥3.08%) versus lower (<3.08%) baseline levels of activated natural killer T cells were associated with longer PFS in lefitolimod-treated patients^58^.

The intratumoral TLR9 agonist tilsotolimod (IMO-2125) induces increases in IFN-γ and IFN-responsive genes within 24 hours of dosing^59^. Tilsotolimod plus ipilimumab was evaluated in the phase 1/2 ILLUMINATE-204 study (NCT02644967), where it was shown to exhibit promising efficacy in the same patient population. Among the 49 patients who received the recommended phase 2 dose (8 mg) and who were evaluable for efficacy in ILLUMINATE-204, the overall response rate (ORR) was 22%, including two CRs, with regression seen in injected and non-injected lesions^60^. Although the combination regimen was tolerable, with no AEs leading to treatment discontinuation or death, almost half (48% [30/62)) of all patients experienced grade 3–4 AEs, most commonly liver enzyme increases and colitis. In the phase 1b, ILLUMINATE-101 study (NCT03052205), which enrolled patients with solid tumors, the most frequent treatment-related AEs associated with single-agent tilsotolimod were pyrexia, fatigue, chills, nausea, and vomiting^61^. The phase 3 ILLUMINATE-301 study (NCT03445533), evaluated tilsotolimod plus ipilimumab versus ipilimumab alone in patients with PD-1 inhibitor-refractory advanced melanoma, however the study was terminated due to lack of efficacy. Tilsotolimod is also being evaluated in combination with ipilimumab plus nivolumab in patients with solid tumors, including microsatellite-stable CRC and melanoma (NCT03865082 [ILLUMINATE-206], NCT04270864 [PRIMO]); in combination with investigational immune checkpoint inhibitors in patients with recurrent/metastatic SCCHN (NCT04196283); and as monotherapy in patients with melanoma (NCT04126876; INTRIM).

Vidutolimod/CMP-001 is a virus-like particle containing a TLR9 agonist that induces the up-regulation of IFN-alpha in plasmacytoid dendritic cells^62,63^ and the production of IFN-inducible genes in T cells and natural killer cells^62^. It is a Class A oligonucleotide^61^. Intratumoral CMP-001 is being investigated as monotherapy and in combination with pembrolizumab in patients with PD-(L)1 inhibitor-refractory advanced melanoma in an ongoing, dose-escalation, phase 1b study (NCT02680184). In an interim analysis, 7 of the 40 patients administered single-agent CMP-001 for 7 weeks developed PRs, corresponding to an ORR of 18%^64^. Among the 98 patients who received CMP-001 (at a polysorbate-20 concentration of 0.01%) plus pembrolizumab for 7 weeks, a total of 23% (*n* = 23) responded (complete response [CR], *n* = 7; PR, *n* = 16). Notably, in patients who had previously progressed on anti-PD(L)1, CMP-001 plus pembrolizumab reduced the size of injected *and* non-injected tumors by ~50%. The most common treatment-related AEs in both treatment arms were flu-like symptoms; injection site reactions were also reported in patients receiving the combination regimen. In patients administered CMP-001 alone or in combination with pembrolizumab, the most common treatment-related grade 3 or 4 AE was hypotension (5% (*n* = 2) and 7% (*n* = 7), respectively). Importantly, no patient died due to a treatment-related AE.

In a recently completed, investigator-initiated, phase 2 study (NCT03618641), neoadjuvant treatment with CMP-001 plus nivolumab was assessed in 30 patients with stage IIIB–D melanoma. After 7 weekly doses, pathologic responses were observed in 70% (21/30) of clinical trial participants (pathologic CR [pCR], 50% (*n* = 15); major pathologic response [MPR], 10% (*n* = 3); pathologic PR, 10% (*n* = 3))^65^. In biomarker analyses, pCR/MPR was shown to be associated with a greater influx of intratumoral plasmacytoid dendritic cells and CD8-positive T cells. Infusion-related grade 3 or 4 AEs occurred in three patients, two of whom discontinued CMP-001.

Additional studies of CMP-001 in combination with approved or investigational immune checkpoint inhibitors are planned or underway in patients with melanoma (NCT04698187, NCT04695977, NCT04401995, NCT04708418, NCT03618641, NCT04401995), recurrent/metastatic SCCHN (NCT04633278), metastatic CRC (NCT03507699), metastatic pancreatic cancer and non-melanoma advanced solid tumors (NCT04387071), patients with castration resistant prostate cancer (NCT05445609), and Merkel cell carcinoma (MCC), cSCC and triple negative breast cancer (NCT04916002). A subcutaneous formulation of CMP-001 is also being explored. A study using the subcutaneous administration of 5 mg, once for 2 weeks followed by intratumoral injections once weekly for 3 weeks followed by every 3 weeks thereafter, in combination with atezolizumab in 29 patients with NSCLC who had progressed s following PD-1 treatment was stopped due to lack of response^66^.

Cavrotolimod (AST-008) is a spherical synthetic oligonucleotide undergoing evaluation in a phase 1b/2 study (NCT03684785) of patients with advanced solid tumors. Although this study was terminated (due to administrative reasons), the phase 1b portion of the study completed enrollment. Patients received single agent cavrotolimod or cavrotolimod plus pembrolizumab. ORR in the overall cohort was 21% (4/19), with responses observed in two patients with MCC and two with melanoma^67^. Three of the four responders had experienced disease progression on anti–PD-(L)1 therapy before study enrollment. Like tilsotolimod and CMP-001, cavrotolimod induced the regression of injected and non-injected tumors. The most frequently reported AEs were injection site reactions and flu-like symptoms. With regard to pharmacodynamics, cavrotolimod (alone and in combination with pembrolizumab) was associated with dose-dependent increases in various cytokines (eg, IP-10) and in the numbers of T cells infiltrating injected and non-injected lesions^68^.

The development of SD-101, a Class C oligonucleotide^61^, was discontinued by its Sponsor in 2019 following a restructuring event^69^. The decision was strategic, as SD-101 in combination with pembrolizumab exhibited promising anti-tumor activity in the phase 1b/2 SYNERGY-001/KEYNOTE-184 study (NCT02521870) of PD-(L)1 inhibitor-naive patients with metastatic melanoma or recurrent/metastatic SCCHN. In the subgroup of patients with metastatic melanoma, an ORR of 76% was observed in those receiving SD-101 2 mg and 49% in those receiving SD-101 8 mg^70,71^. Responses were seen in injected and non-injected lesions, as well as in PD-L1–positive and PD-L1–negative tumors. Among patients with recurrent/metastatic SCCHN, an ORR of 22% was reported in those administered SD-101 2 mg and 26% in those administered SD-101 8 mg^72,73^. Treatment with SD101 and pembrolizumab precipitated an influx of T cells in both tumor types, and the most common SD-101–associated AEs were injection site reactions and flu-like symptoms^71,73^.

Clinical development of SD-101 continues in trials and is currently being evaluated in combination with nivolumab and radiation therapy in patients with chemotherapy-refractory metastatic pancreatic cancer (NCT04050085); in combination with pembrolizumab, intermittent androgen deprivation therapy, and stereotactic body radiation therapy in patients with newly diagnosed, hormone-naive, oligometastatic prostate cancer (NCT03007732); as neoadjuvant therapy in combination with pembrolizumab in patients with breast cancer (NCT01042379; I-SPY); in combination with nivolumab and ipilimumab in patients with uveal melanoma (NCT04935229); in combination with pembrolizumab or nivolumab and ipilimumab in patients with liver tumors (NCT05220722); in combination with BMS986178 in patients with solid tumors (NCT03831295).

## Conclusion

**Fig. 2:**
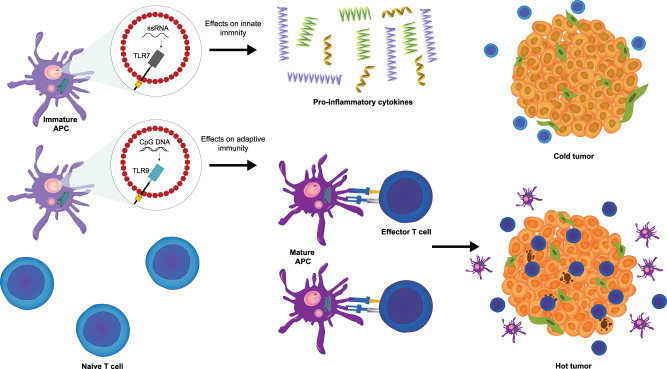
Hypothesized mechanism for synergism between TLR agonists and immune checkpoint inhibitors in enhancing antitumor immunity. TLRs are involved in both innate and adaptive immunity. It is hypothesized that the anti-tumor effects of TLR agonists are likely attributable to their ability to convert “cold tumors” into “hot tumors”, as pharmacodynamic analyses have demonstrated the ability of these agents to induce the expression of pro-inflammatory cytokines and to stimulate the influx of effector T cells into tumor tissue.

Given the potential to both stimulate and enhance anti-tumor immunity, as well as the number of planned/active clinical studies, it is apparent that clinicians see promise in the use of TLR agonists to treat cancer. Based on the preliminary data available, TLR7 and TLR9 agonists currently in development suggest anti-tumor activity when used as monotherapy or in combination with approved immune checkpoint inhibitors. Notably, objective responses have been reported in patients with PD-(L)1 inhibitor-resistant disease treated with CMP-001 ± pembrolizumab or cavrotolimod ± pembrolizumab and in those with PD-L1–negative tumors treated with SD-101 plus pembrolizumab. Given the role of TLRs in both innate and adaptive immunity, the anti-tumor effects of TLR agonists are likely attributable to their ability to convert “cold tumors” into “hot tumors”, as pharmacodynamic analyses have demonstrated the ability of these agents to induce the expression of pro-inflammatory cytokines and to stimulate the influx of effector T cells into tumor tissue (Fig. 2). In terms of safety, the most common AEs associated with TLR agonists appear to be flu-like symptoms, injection site reactions, fatigue, and decreased leukocyte counts (e.g., lymphocytes, neutrophils). Importantly, when used as part of a combination regimen, investigational TLR7 and TLR9 agonists, such as CV8102, tilsotolimod, and CMP-001, do not appear to increase the toxicity of approved immune checkpoint inhibitors. This is notable as other TLR agonists, such as IMO-2055, have been discontinued owing to an increased risk of AEs when used as part of combination therapy^74^.

Given the conflicting roles of different TLRs and of the same TLR in different tumor types, novel clinical study designs, namely adaptive designs, may be of value in efficiently evaluating the efficacy and safety of novel TLR agonists. Adaptive study designs allow for smaller numbers of patients to be assessed and/or for shorter evaluation periods relative to traditional clinical trial designs^75^. Once preliminary data become available, informed decisions can be made on whether assessment of a specific tumor cohort and/or treatment regimen should be abandoned or expanded. Biomarkers, such as those identified in the translational research setting, tend to underpin adaptive study designs, as they can inform patient selection (e.g., those with HER2-positive tumors) or be used to measure treatment response.

There is precedent for the use of adaptive study designs in the oncology setting, including MyPathway (NCT02091141), a multiple-basket, phase 2a study evaluating the clinical potential of approved targeted agents in non-approved tumor types; the ongoing phase 1/2 study (NCT03416335) of DSP-0509 ± pembrolizumab in those with advanced solid tumors; the phase 2 AGADIR study (NCT03915678), which is assessing atezolizumab plus BDB001 and radiotherapy in various tumor types, including pancreatic cancer and PD-(L)1 inhibitor-refractory NSCLC and bladder cancer; and the phase 1 I-SPY study (NCT01042379), which is studying approximately 20 different regimens for the neoadjuvant treatment of breast cancer. As the anti-tumor activity of TLR agonists has been correlated to the proliferation of dendritic cells and lymphocytes, this may serve as a suitable biomarker of clinical activity in adaptive studies. Given the positive signals stemming from preliminary analyses, more robust efficacy and safety data are eagerly anticipated from ongoing studies of TLR agonists in development for the treatment of solid tumors.

## Data Availability

No datasets were generated or analyzed for this article.
